# Estimation of neuronal task information in fMRI using zero frequency resonator

**DOI:** 10.1016/j.neuroimage.2023.119865

**Published:** 2023-01-05

**Authors:** Sukesh Kumar Das, Anil K. Sao, Bharat B. Biswal

**Affiliations:** a Indian Institute of Technology Mandi, Mandi, HP 175005, Himachal Pradesh, India; b Indian Institute of Technology Bhilai, Bhilai, Chhattisgarh 492015, Chhattisgarh, India; c New Jersey Institute of Technology, Newark, NJ 07102, New Jersey, USA

**Keywords:** fMRI, Functional connectivity, Point process analysis, Temporal BOLD event estimation, Zero frequency resonator, Conditional rate map

## Abstract

In functional magnetic resonance imaging (fMRI), temporal onsets of BOLD events contain crucial information on activity-inducing signals and make a significant impact in the analysis of functional connectivity (FC). In literature, the estimation of the onsets of the BOLD events from the acquired blood oxygen level-dependent (BOLD) signal using fMRI is mostly performed by choosing locations with a high value of the BOLD signal. This approach may give false onset points because it can incorporate redundant onsets which could be due to non-neuronal activity or can exclude true low-valued BOLD signals. In this study, we present a novel approach to estimating the temporal onsets of the BOLD events using a zero frequency resonator (ZFR) without necessitating information regarding the experimental paradigm (EP). The proposed approach exploits the impulse-like characteristic of activity-inducing signal to estimate the temporal onset points of BOLD events using ZFR which has been widely studied in the area of speech signal processing to estimate the glottal closure instances. The idea behind the approach is that an ideal neuronal impulse has, in principle, equal energy at all frequencies, including around the zero frequency, and will preserve the information of the temporal onsets of the BOLD events at its output. The ZFR-based approach estimates two important features, namely: 1) task-induced temporal onsets of the BOLD events in the fMRI time course and 2) high SNR (HSNR) regions around the estimated BOLD events. Both the estimated features are used to obtain the FC. Results are demonstrated using both the synthetic and experimental (event-related finger tapping and block design working memory) data. We show that a small number of plausible time points, estimated by ZFR, can convey sufficient information indicating the associated activation pattern. The method also illustrates its significance over the conventional correlation and threshold-based conditional rate analysis to estimate FC. The study demonstrates that ZFR-estimated BOLD events and HSNR regions can produce sufficient functionality of the brain in the task paradigm.

## 1. Introduction

Functional magnetic resonance imaging (fMRI) measures changes in oxygenation and deoxygenation of red blood cells and implicitly reflects the neuronal activity under different task conditions or during rest (Ogawa et al., 1990). Due to its non-invasiveness in addition to high spatial and temporal resolution, fMRI has become a popular method for studying systems-level neuroscience. It is widely used to investigate the human brain’s sensory, motor, and cognitive functions. A current hypothesis is that performing a task results in increased neuronal firing in eloquent regions of the brain. This increase in neuronal firing causes more oxygen consumption leading to increased blood flow and consequently an increase in fMRI signal during task responses. Thus, the measured blood oxygen level-dependent (BOLD) signal, in fMRI, for a specific voxel represents the local neuronal activity in the brain and is an indirect measure of neuronal states over time. The BOLD response (*m*[*n*]) is postulated as the result of convolution of the neuronal activity with the hemodynamic response function (HRF) (Wu et al., 2013) and can be written as:

(1) m[n]=s[n]∗h[n]+ϵ[n],

where *n* and * denote the time index and convolution operation, respectively. The HRF, denoted as *h*[*n*], and *ϵ*[*n*] is the noise evoked during measurement. *s*[*n*] denoted the underlying neuronal activity signal (NAS) which carries timing and intensity information of neuronal events in a BOLD time course. Hypothetically, it is modeled as a train of impulse functions (Glover, 1999; Wu et al., 2013). The temporal onsets of the impulse are called neuronal events which are correlated with the external stimulus for task-fMRI. In the remaining part of this manuscript, the term NAS has also been referred to as neuronal-related or activity-inducing signal (AIS) in previous literature for example in Caballero Gaudes et al. (2013); Gaudes et al. (2011); Karahanoğlu et al. (2013); Uruñuela et al. (2022).

The underlying neuronal activity governs functional connectivity (FC) in the brain. Estimation of the FC in the brain, using the observed BOLD signal may not be precise because the NAS has a characteristic of impulses in the time domain and gets a sluggish modulation by HRF in the acquired BOLD signal. In practice, both the NAS (*s*[*n*]) and HRF (*h*[*n*]) are typically unknown, thus their estimation from the observed *m*[*n*] is an ill-posed problem. Several deconvolution methods have been proposed in the literature to estimate the neuronal activity (Aggarwal et al., 2015; Bush and Cisler, 2013; Buxton et al., 1998; Caballero Gaudes et al., 2013; Das et al., 2021; David et al., 2008; Friston et al., 2000; Gaudes et al., 2011; Glover, 1999; Karahanoğlu et al., 2013; Seghouane and Shah, 2012; Sreenivasan et al., 2015; Wink et al., 2008; Wu et al., 2013). Most of these methods assume either parametric (Bush and Cisler, 2013; Caballero Gaudes et al., 2013; Gaudes et al., 2011; Karahanoğlu et al., 2013) or non-parametric (Das et al., 2021; Glover, 1999; Sreenivasan et al., 2015; Wink et al., 2008) model of HRF for the estimation of NAS. In the parametric approach, the shape of HRF is modeled by a few parameters, which could be susceptible to overfitting, and remains unclear if these models accurately reflect the biophysical process Sreenivasan et al. (2015). These methods include the estimation of the HRF and then the deconvolution of it from the observed signal. Traditional deconvolution approaches described in Glover (1999); Sreenivasan et al. (2015); Wink et al. (2008) have a non-parametric assumption on the HRF model and require the information on an experimental paradigm (EP). The method proposed by Sreenivasan and colleagues (Sreenivasan et al., 2015), along with the information of EP also exploits the observation that HRF belongs to mostly the low-frequency region in the spectrum of the BOLD signal. The method uses homomorphic filtering which converts the BOLD time course into cepstrum domain and separates the NAS by selecting a suitable cutoff quefrency. In several of these methods (Bush and Cisler, 2013; Glover, 1999; Seghouane and Shah, 2012; Sreenivasan et al., 2015; Wink et al., 2008), the estimation of neuronal information in the form of the NAS includes the contribution of EP. However, it may be difficult to exactly follow an EP in clinical cases including stroke or traumatic injury, the subjects may not be able to perform according to the stimulus presentation. In schizophrenia subjects experiencing a hallucination, the subject’s perception may switch over time (Gaudes et al., 2011). Even in healthy subjects, for a number of tasks that may have habituation or learning effects using a standard EP model may not be optimal. Such an issue can be addressed by estimating the precise temporal onsets of BOLD events. These events eventually carry information on neuronal activation with some delays. Point process analysis (PPA) is a straightforward way using which one can estimate the onsets.

We have earlier discussed the deconvolution methods and the possible difficulties in estimating NAS. Now, embarking on the PPA, we focus on the temporal onsets of the BOLD events which are the consequences of neuronal firing for the exposure of the external stimulus. The PPA has been extensively studied to estimate FC in resting state fMRI (Cifre et al., 2017; Li et al., 2014; Liu and Duyn, 2013; Rolls et al., 2021; Tagliazucchi et al., 2012;^2011^; ^2016^; Wu et al., 2013; Wu and Marinazzo, 2015; Zhuang et al., 2018). Recently, Freitas and colleagues considered the onset of the BOLD events obtained using high values of BOLD signal and combined it with external stimulus to form Psychophysiological Interactions (PPI) (Freitas et al., 2020). The supra thresholded time frames (image volumes), selected using PPI, were then used for static and dynamic analysis of FC in visual task-fMRI. The experimental results demonstrated that only selected time points, instead of the entire time periods of the BOLD signal, are sufficient to estimate the FC without significant loss of precision. The method, however, requires information on the task paradigm and selection of the optimal threshold which may be challenging.

Temporal onsets of the neuronal events are associated with high-valued samples in the BOLD time course and are sufficient to represent the temporal response of neuronal activity at voxels. Now, following the premise that the NAS has the characteristic of impulses and only a few temporal onset points can derive the FC, in this paper, we propose an approach based on zero-frequency resonator (ZFR) (Yegnanarayana and Murty, 2009) to extract two features to estimate FC in task-related fMRI. These features are: (1) estimated temporal onsets of BOLD events in the time course and (2) temporal high SNR (HSNR) regions around the estimated events. The proposed approach exploits the impulse-like characteristics of neuronal activity in the BOLD time course and BOLD events are estimated using the ZFR for task-fMRI data. It is widely studied in speech signal processing to extract the instant of glottal closure (Murty et al., 2009; Prasanna and Pradhan, 2011; Yegnanarayana and Murty, 2009). The ZFR exploits the fact that an ideal impulse has, in principle, the equal amplitude at all frequencies, including around the zero-frequency in the spectrum. Thus, the information of the BOLD events will be preserved in the output of the ZFR while the substantial effect of the HRF and noise acquired during the measurement will be mitigated. The extracted features, namely estimated onsets of BOLD event and HSNR regions are used to obtain the FCs using conditional rate (CR) and HSNR correlation, respectively. Experimental analysis has demonstrated that even a few time points (less than 7%), extracted by ZFR, can estimate the respective conditional rate map (CRM). The results also underpin that HSNR regions in time courses can produce correlation maps using less than 43% of the information, instead of entire voxel time courses. It should also be noted that the proposed approach does not require the information of the task paradigm to extract the onsets of the BOLD event in the fMRI time course for the estimation of FC. Here the hypothesis is that the NAS will be reflected at all frequencies while the non-parametric approach of deconvolution in Sreenivasan et al. (2015) assumes that the NAS has predominantly high-frequency components in comparison to the HRF. Thus, there may be a possibility that it may include a noise component. In this study, we have demonstrated the results using synthetic fMRI data and fMRI acquired for event-based finger tapping and complex block working memory task data.

## 2. Method and materials

### 2.1. Method

**Fig. 1.**
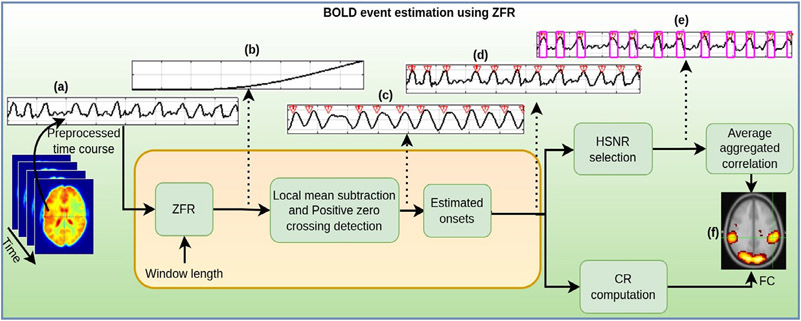
Estimation of FC using the BOLD event extracted by ZFR in task fMRI: (a) A preprocessed time course taken for the left primary motor area, (b) output of the ZFR. It grows approximately as a polynomial function of time, (c) zero frequency filtered signal after local mean subtraction. Positive zero crossings indicate the temporal onsets of the BOLD events, (d) estimated temporal onsets and the fMRI time course, (e) HSNR segments in the BOLD time course, and (f) functional connectivity using CR or HSNR correlation map.

Estimation of underlying neuronal information involved in the task in the form of temporal onsets of the BOLD events using ZFR and computation of FCs are illustrated using a schematic diagram in Fig. 1. ZFR has been extensively studied in the area of speech signal processing (Yegnanarayana and Murty, 2009). The method exploits the impulse-like characteristics of excitation during the production of voiced speech. Narrowband filtering of the speech signal at zero-frequency provides evidence of the temporal point around excitation by minimizing the impact of the time-varying vocal tract system. Extending the analogy of speech production mechanism to fMRI, the observed BOLD time course, defined in Eq. 1, can be considered as the output of the hemodynamic system (*h*[*n*]) for neuronal excitation (*s*[*n*]), which possesses impulse-like characteristics. Thus, the output of the ZFR for a time course will preserve mostly the information of the temporal onsets of the BOLD events due to the transient behavior of the neuronal activity and the effect of the HRF and noise would be highly suppressed. An ideal zero-frequency resonator is a second-order system whose system function is given by (see appendix A)

(2) $$H_{z}(z)=\frac{Y(z)}{M(z)}=\frac{1}{1-2z^{-1}+z^{-2}},$$

where *Y*(*z*) and *M*(*z*) are the *z*-transform of output *y*[*n*] and input *m*[*n*] of a ZFR respectively. Thus the output of the ZFR for a given input (*m*[*n*], BOLD time course) will be

(3) y[n]=m[n]+2y[n−1]−y[n−2].

The recurrence relation can be interpreted as the cumulative sum of the input performed twice. Thus, the output of ZFR for a BOLD time course will grow approximately as a polynomial function of time. But the fluctuations in the output contain the information of BOLD events (temporal). In order to extract the information, the local mean is subtracted from the output signal (Yegnanarayana and Murty, 2009) and we call it zero frequency filtered signal. The resulting signal is given by

(4) $$z[n]=y[n]-\frac{1}{2N_{1}+1}\sum_{k=-N_{1}}^{N_{1}}y[n-k],$$

where (2*N*_1_ + 1) is the size of the window in samples. In the filtered signal (*z*[*n*]), the positive zero-crossings where the signals change their sign from negative to positive correspond to the timing of the onsets of the BOLD event.

**Fig. 2.**
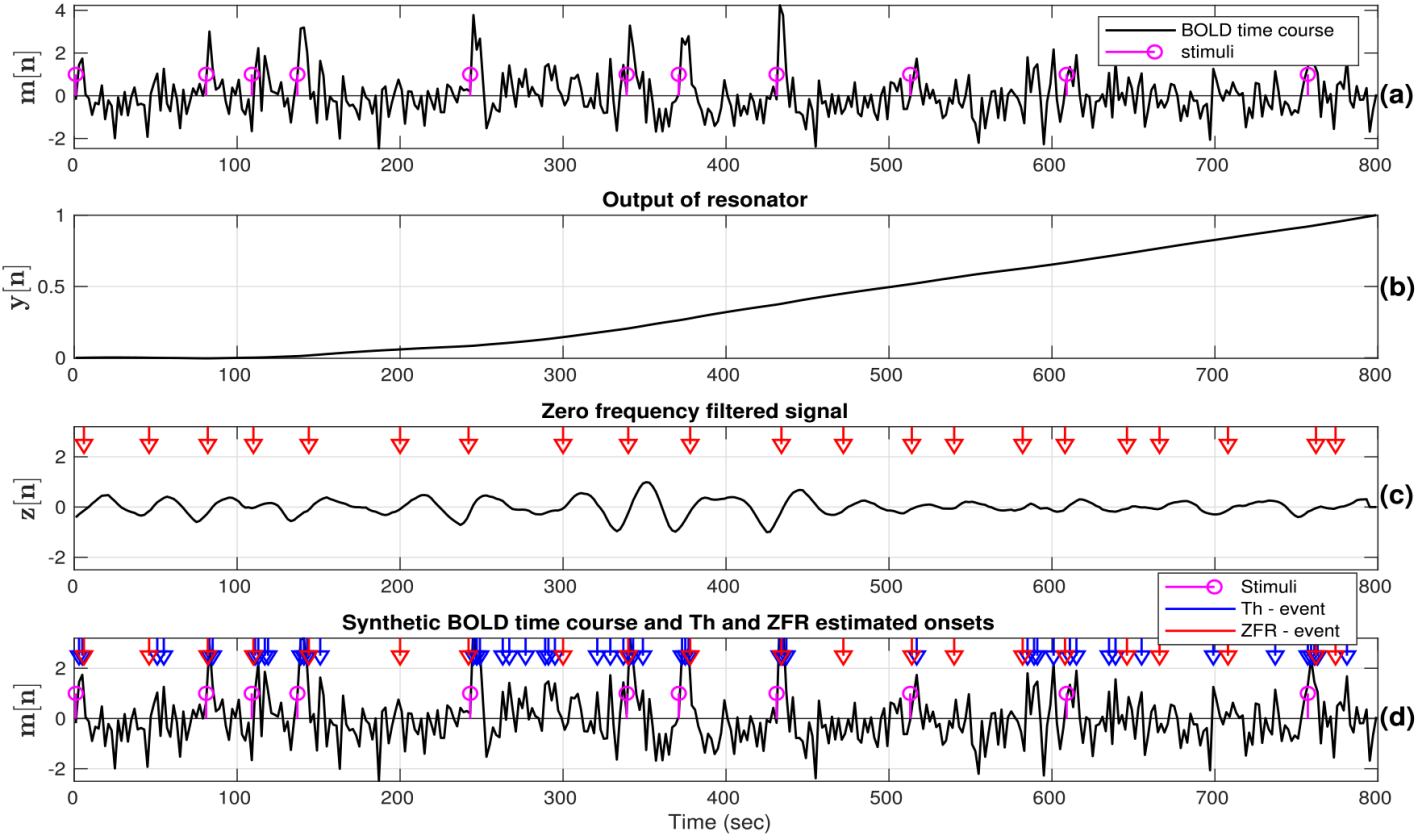
Output of the steps involving the ZFR for a synthetic BOLD response (TR=2 s, 400 samples) : (a) Actual onset locations (magenta stems) and synthetic BOLD time course, (b) output of the resonators (growing), (c) ZFR output after local mean subtraction and estimated onsets (red markers) at positive zero crossing. The window length for the synthetic data is *N*_1_ = 15, (d) synthetic BOLD time course, stimuli timing (stems) and onset locations (red markers) estimated by ZFR, and thresholding (blue markers).

The efficacy of the ZFR to estimate the timing of the BOLD events for a synthetic fMRI time course is illustrated in Fig. 2. A sequence of the unit sample at random locations was generated (stems in 2(a)) and convolved with the canonical HRF (obtained from SPM12) to generate a synthetic BOLD time course with 400 samples and TR of 2 s. Random white noise was added to the resultant signal (see Fig. 2(a)). The range of the values of the output of the ZFR is very large because of the cumulative sum, as shown in Fig. 2(b). The local mean subtracted signal (*N*_1_ = 15) has been shown in Fig. 2(c) and the red markers indicate the positive zero crossings which give the discontinuities due to the neuronal excitation in *m*[*n*]. Timings of the estimated onsets by using ZFR, are shown in Fig. 2(d) (red marker). We have also illustrated the estimation of the temporal onsets of the BOLD events using a threshold approach in Fig. 2(d) (blue marker). Here, the time course (*m*[*n*]), after z-score normalization, crossing the threshold value 1 was marked as the BOLD events (Tagliazucchi et al., 2012; Wu et al., 2013). It can be observed that there are several false detections (matching within the interval of 2TR on the right side of the stimuli) of BOLD events using the threshold method in comparison to ZFR. The false negatives may increase with increasing the level of noise. The onsets obtained using the threshold method cannot always be associated with the actual location of impulses employed while generating a synthetic BOLD signal.

#### 2.1.1. Features associated with ZFR output

The ZFR method is used to estimate the temporal onsets of the BOLD events in the fMRI time courses. The onset time points were used to compute conditional rate (CR) (Liu and Duyn, 2013; Liu et al., 2018; Tagliazucchi et al., 2012), a useful metric to estimate the FC. The CR is computed using the ratio of the number of estimated onsets in the target voxel-time course, matched (with at most temporal delays of two units) with the seed voxel-time course, to the total number of onsets present in the seed voxel time course. If the seed and target voxels are co-activated then their neuronal events will match well and the value of the CR will be close to 1. On the contrary, if the CR value is low then the seed and target voxels are not co-activated. The second feature, temporal HSNR regions, i.e. the BOLD values around the estimated events, contains most of the energy ensuring the presence of the BOLD responses due to neuronal excitation in the fMRI time course. Now, this feature is denoted in terms of *n*_*c*_ (location of onset). Then the HSNR region will be estimated as *m*[*n*_*c*_ − 2, *n*_*c*_ + *w* − 3], where *w* indicates the length of the HSNR window (we consider *w* = 6 in our study). The temporal region, around the onset, is called the ‘HSNR region’ because high activity reduces the effect of noise in the observed fMRI time course. The selected HSNR segments of the seed voxel time course and the same temporal regions in target time courses were used to compute Pearson’s correlation coefficient and an average of its aggregation over all the selected HSNR regions is used to quantify the co-activation strength.

### 2.2. Data description and standard preprocessing

The synthetic fMRI time courses were simulated for 5 subjects using the publicly available SimTB toolbox implemented in Matlab (Erhardt et al., 2012). A total of 150 time points were acquired with a TR of 2 s. Every 2D brain slice is with voxel dimensions 100 × 100. Two spatial components (equivalent to right and left auditory) were selected and then inter-subject variability was introduced by spatial variability in translation, rotation, and spread. Only standard tone type events with probability 0.07 are convolved with canonical HRF to generate component-time courses. Voxelwise time courses are generated as a linear combination of spatial components weighted by the component-time courses with added baseline tissue weights. Furthermore, Rician noise was added to the simulated data with random contrast-to-noise ratios (CNR) ranging from 0.65 to 1.0 for different subjects.

The finger tapping data were acquired from 5 subjects (2M/3F, average age of 24 years) on a 3T General Electric (GE) MRI scanner with an eight-channel coil Discovery MR750 in two sessions. Two types of visual stimuli, periodic and aperiodic stimuli, were presented on the screen to instruct the subjects to tap their fingers. Acquired anatomical data contains 156 slices i.e. one 256 × 256 × 156 volume. For periodic stimuli, a total of 187 and 213 functional volumes were acquired in sessions 1 and 2 respectively. Again, data by presenting the aperiodic stimuli were collected with 175 and 199 volumes in two sessions respectively. Other scanning parameters are as follows: Echo time (TE) = 0.03s; repetition time (TR) = 2s; field of view = 240 × 240*mm*^2^, flip angle = 90°; slice thickness = 3; matrix size = 64 × 64; # slices = 42.

The block design working memory data was acquired from 19 healthy subjects (8 M/11 F, average age of 23 years) on a 3T GE Sigma scanner with an eight-channel head coil. Within each run, there were 4 blocks each with 8 trials. For each trial, the stimuli were presented for 0.5s with an inter-stimulus interval of 2.5s. The *n*-back task tested working memory of the location of letter stimuli. A white fixation cross was presented in the center of the screen on a dark background. On each trial, a random letter was presented in 1 of the 4 visual field quadrants around the fixation. In a *n*-back condition (*n* = 1 or 2), subjects were asked to press the left button with the left thumb when the location of the current letter matched with the location of the letter presented *n* items back, and to press the right button with the right thumb when the locations did not match. One-third of the total trials were “matches”. The orders of the trials were counterbalanced across participants. The participants were instructed to focus only on the location of the letter not on the letter itself and to classify the stimuli as accurately and quickly as possible. Visual stimuli were presented, and responses were collected using E-Prime (Psychology Software Tools). More details regarding the experimental paradigm can be found in a recently published paper (Di et al., 2020). The working memory data were acquired using the following scan parameters: TR = 2s; TE = 30 ms; field of view = 240 × 240*mm*^2^; flip angle = 90°; # slices (axial) = 42; slice thickness = 3*mm* with gap = 0; matrix size = 64 × 64; #images (block design run) = 113. T1-weighted images were acquired using the following scan parameters: TR = 0.006s; TE = minimum; field of view = 256 × 256*mm*^2^; flip angle = 12°; # slices (sagittal) = 156; slice thickness = 1 mm.

The event-related and block design of both data were pre-processed using SPM12 ^1^ software with MATLAB R2019a. The initial 3 time points of data from all fMRI scans across subjects were discarded to get a steady magnetic field. Anatomical images were reoriented with respect to the Montreal Neurological Institute (MNI) space and functional images were reoriented with respect to the anatomical images. Functional images were then realigned followed by slice time correction. Anatomical images were co-registered with functional images. Anatomical images were then segmented into gray matter (GM), white matter (WM), and cerebrospinal fluid (CSF). Functional images were then normalized to MNI space using the deformation field obtained in segmentation. The normalized functional images were spatially smoothed with a Gaussian kernel of FWHM 5*mm* × 5*mm* × 5*mm*. The size of the window for ZFR in BOLD fMRI analysis is empirically chosen as 15 and 11 samples for the event and block design data respectively.

## 3. Experimental results

The usefulness of the two ZFR-based features, estimated temporal onsets of the BOLD events and BOLD signals around HSNR regions, are demonstrated by computing the CR and HSNR correlation respectively. The FC is then estimated using the seed-based approach. To investigate the spatial extents of activation due to a task performance using the proposed method, the canonical HRF (from SPM) was convolved with the task reference function, and voxel-wise correlation was performed for every voxel time course in the brain using the convolved signal. This process was repeated for all the subjects and an average correlation map is demonstrated. The voxel-wise onsets were estimated by the threshold (crossing the threshold value of 1 in standardized time course marked as an onset Tagliazucchi et al. (2012); Wu et al. (2013)) and ZFR-based approaches. Following the estimation of onsets, CRs are computed for every voxel (target) across the brain using the reference function (convolved) and CRMs are obtained for every subject. The average CRM of all the subjects is computed using a threshold-based approach and ZFR-based approach, respectively. Further, FC is obtained by averaging the aggregated correlation between HSNR regions at convolved time course and the same temporal regions in the target voxel time course for the experimental data. The average of the aggregated correlation, for every voxel using the convolved signal, is computed across the brain, and finally, the average map of all subjects leads to an HSNR correlation map. The top 20% of the voxels were considered after performing min-max normalization, for each of the considered methods. After thresholding, we considered clusters of voxels that are connected with at least 16 (for synthetic data it is 8) active neighborhood voxels. The entire procedure is also repeated using an actual seed time course taken from the left Primary motor area (−53, 0, 22) and FCs are obtained.

### 3.1. FC using synthetic data

**Fig. 3.**
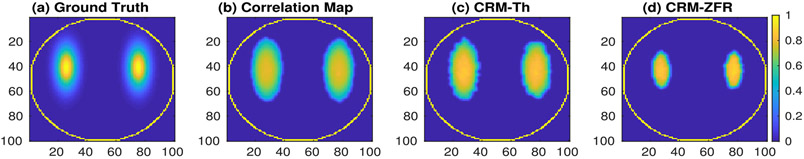
FC in synthetic data: (a) Ground truth (two spatial components equivalent to right and left auditory components), estimated maps using (b)correlation (c) CR with threshold method, and (d) CR with ZFR using an active time course.

The synthetic data generated for 5 subjects were used to estimate FC using correlation, conditional rates with threshold, and the ZFR approach. In Fig. 3, the ground truth and the estimated FCs, using the three methods were shown. It is observed that CRM with ZFR can identify the two auditory (equivalent) components and is a close estimate to the ground truth whereas the other two methods could extend the estimated components (spatial) beyond the actual component regions.

### 3.2. Efficacy of ZFR on event related finger tapping task data

**Fig. 4.**
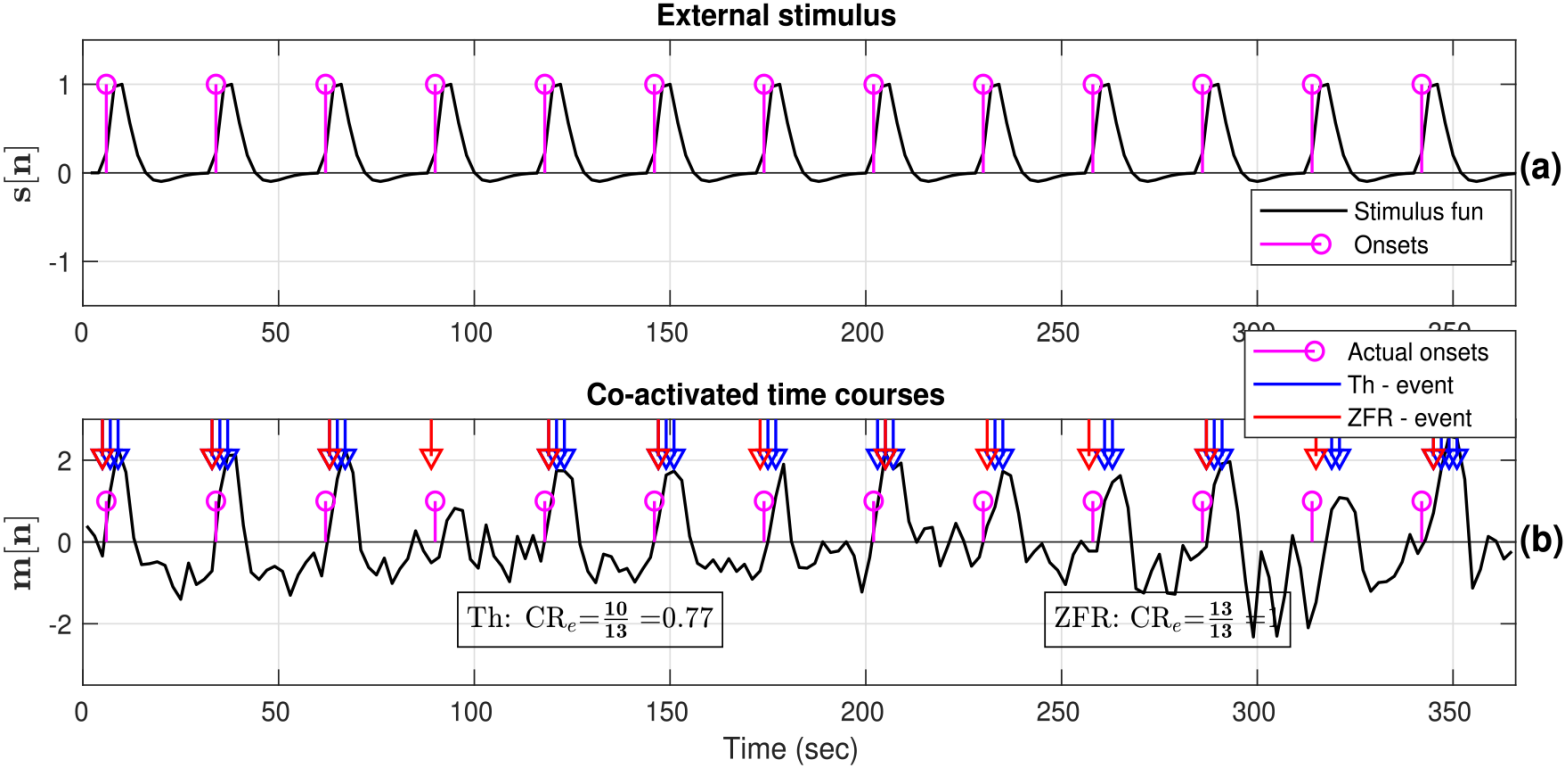
External stimulus and estimated temporal events in the BOLD response: (a) Periodic onsets of the given stimuli (magenta stems) and stimulus function obtained by convolving the onsets with canonical HRF, (b) active seed time course (taken from left motor cortex), actual onsets, and estimated onsets (blue and red marker) using thresholding and ZFR approach respectively. Actual onsets (stimuli timing) at 4, 10, and 12th are not well matched with thresholded onsets, and the rest are well matched. On the other hand, all the estimated onsets by ZFR are well matched with the actual stimuli with a small delay.

**Fig. 5.**
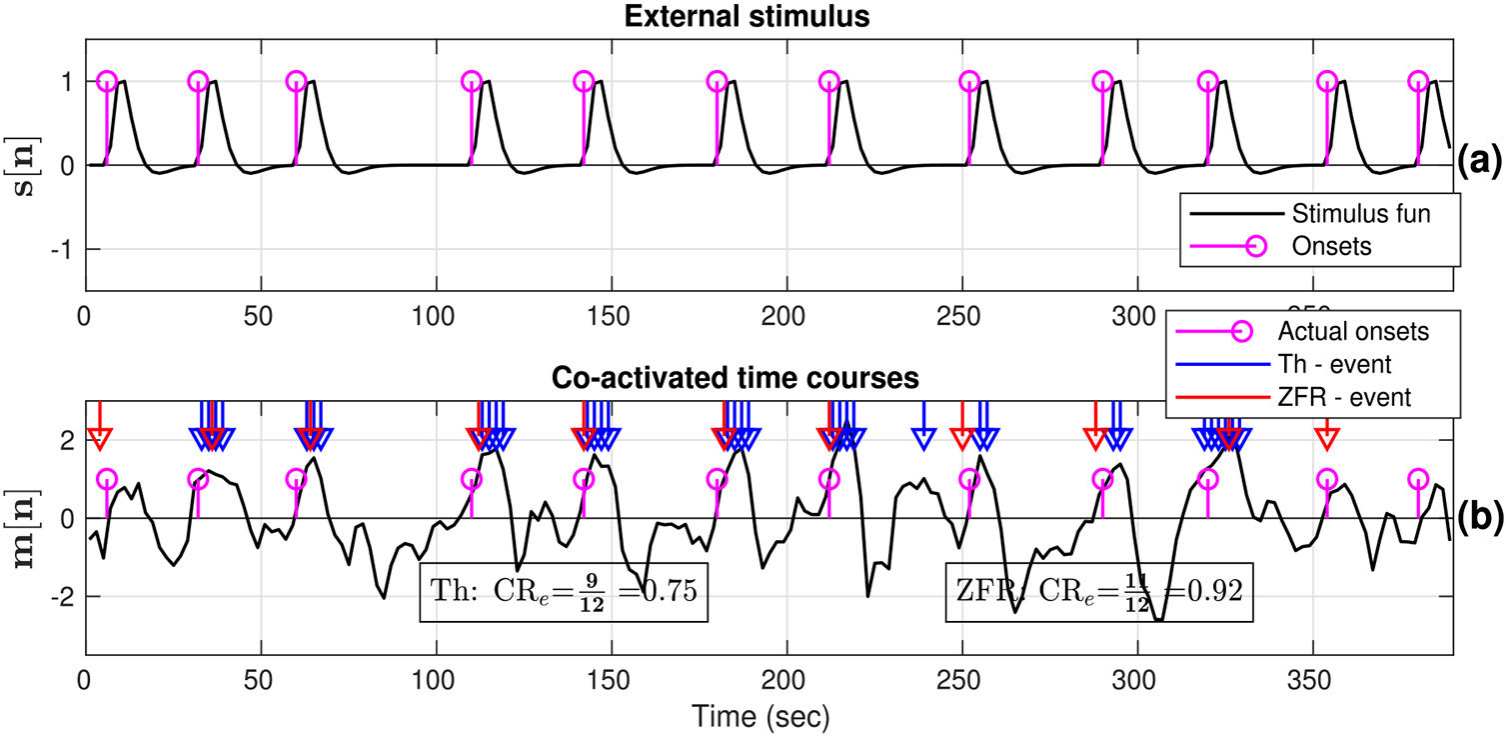
External stimulus and estimated temporal events in the BOLD response: (a) Aperiodic onsets of the given stimuli (magenta stems) and stimulus function obtained by convolving the onsets with canonical HRF, (b) active seed time course (taken from left motor cortex), actual onsets, and estimated onsets (blue and red marker) using thresholding and ZFR approach respectively. Actual onsets (stimuli timing) at 1, 11, and 12th are not well matched with thresholded onsets, and the rest are well matched. On the other hand, all the estimated onsets by ZFR are well matched except the last one with the actual stimuli with a small delay.

For meaningful validation, we consider event-related data where active voxels distinctly elicit the events corresponding to the external stimuli. The proposed method is employed to the finger-tapping data with periodic and aperiodic visual stimuli to study the effectiveness of the ZFR-driven features in estimating FC and to compare it with conventional approaches. The employed periodic and aperiodic stimuli (magenta) are shown in Figs. 4(a) and 5(a) respectively. The actual stimuli are convolved with canonical HRF and resultant signals (black) are shown in Figs. 4(a) and 5(a) for periodic and aperiodic stimuli respectively. The representative (target) time courses for periodic and random stimuli are shown in Figs. 4(b) and 5(b) respectively. The chosen target voxels are from the left motor cortex, as the region will be active due to its involvement in finger tapping task. The temporal events or onset locations are estimated using thresholding and ZFR methods. The estimated onsets are used to compute CR using the external stimuli and denoted as CR_e_. In computing CR_e_, we consider two units of advancements as well as two units of delays of the target signals for counting matches with the stimuli. It was followed with the consideration that there is a few seconds delay between the external stimuli and the BOLD response. The following observations could be made:

- In the ZFR-based approach, the estimated events coincided significantly with the task stimuli, with some delay as there is typically a temporal difference of a few seconds between stimuli presentation and fMRI signal changes due to neuronal excitation. However, we can observe that there are false negative (FN) at 95 sec and false positives (FP) at 263 and 318 sec for periodic stimuli (Fig. 4) and there are FN at 13 sec and FPs at 353 and 392 sec (Fig. 5) in the case of thresholding. On the other hand, FNs are fewer in the number using the ZFR-based method. In the threshold-based approach, the high values of the BOLD signal which were identified as the onset points could not always be associated with the actual stimuli location.
- The threshold and ZFR based CR_e_ values for the periodic stimuli are 0.77 and 1 respectively and for the aperiodic stimuli, they are 0.75 and 0.92 respectively for the given time courses ((Fig. 4(b) and Fig. 5(b) respectively)).
- For both of the stimuli, the observed target time courses are active and the values of the CR_e_s, estimated by ZFR, are greater than that of the threshold method (Fig. 4(b) and Fig. 5(b) respectively). So, the CR using the ZFR-based approach is better than the threshold-based approach.

**Fig. 6.**
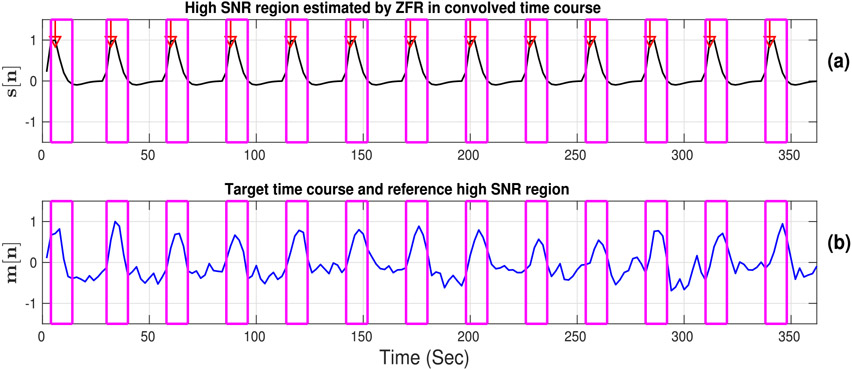
High SNR region: (a) Estimated temporal high SNR regions around the onsets estimated by ZFR in convolved time course, (b) superimposed seed (convolved signal) high SNR regions on target voxel time course.

Fig. 6 illustrates that the high BOLD values in the convolved time course (stimulus function) align with the high values in the target time course and can be a good feature to estimate the strength of co-activation between voxel time courses in task fMRI. Extracted HSNR regions were demonstrated in the reference time course in Fig. 6(a) and the same temporal regions were overlaid on the target time course (Fig. 6(b)).

**Fig. 7.**
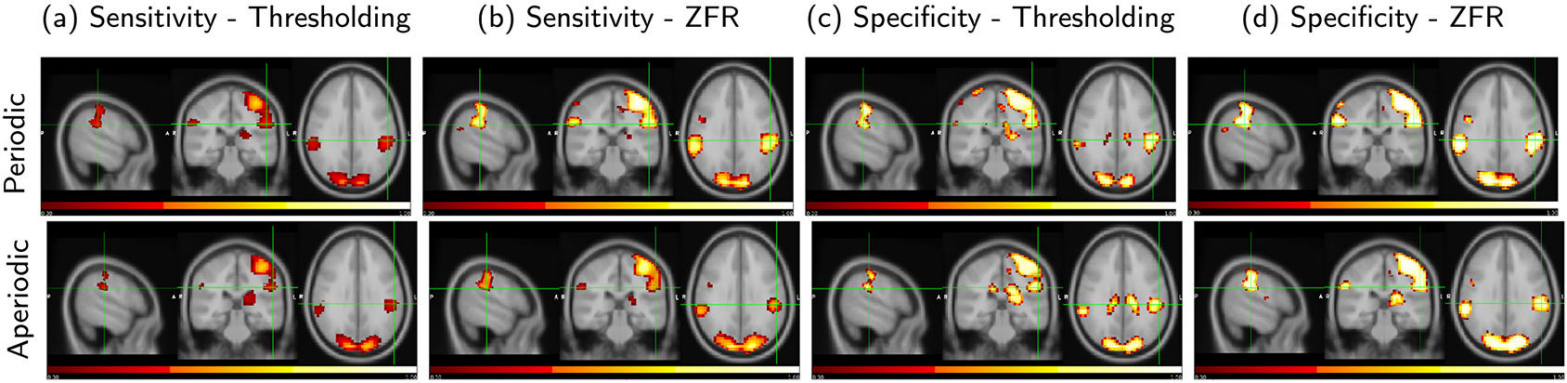
Sagittal, coronal and axial views of average sensitivity and specificity map of 5 subjects using the actual stimulus signal for periodic (top row) and aperiodic stimuli (bottom row): Sensitivity map using the estimated onsets by (a) threshold and (b) ZFR methods respectively, specificity map using the estimated temporal onsets by (c) threshold and (d) ZFR methods respectively. All maps are represented on the same scale (0.3 - 1).

Average sensitivity and specificity of the estimated events using the given stimuli are computed across the voxels in the brain using thresholding and the ZFR-based methods and illustrated in the Table 1. As there is a delay (about 100 ms to 2s) between the presented stimuli and the BOLD response, the estimated onset of the BOLD event is considered to be matched with the external stimuli if it lies within the interval of 2TR on the right side of the stimuli. The detected events are used for computing the sensitivity and the specificity w.r.t. the stimuli, as given by

(5) $$sensitivity=\frac{TP}{TP+FN},$$

(6) $$specificity=\frac{TN}{TN+FP},$$

where True positive (TP) - Stimulus (1) is detected as the temporal BOLD event (1). True negative (TN) - Non-stimulus (0) is detected as the temporal BOLD non-event (0). False positive (FP) - Non-stimulus (0) is detected as the temporal BOLD event (1). False negative (FN) - Stimulus (1) is detected as the temporal BOLD non-event (0). It can be noticed that both the sensitivity and specificity scores are higher in the case of the ZFR than in the thresholding method and it is true for both the kinds of stimuli (periodic and aperiodic). It can also be observed that the specificity score is greater than the sensitivity score because the number of non events is more than the number of events in the BOLD time courses. The average sensitivity and specificity maps of 5 subjects are also demonstrated in Fig. 7. The primary motor and occipital cortices find high sensitivity and specificity scores in the maps as the external excitations are associated with finger tapping with visual stimuli. For both the stimuli (periodic and aperiodic), the ZFR-based sensitivity and specificity maps are more distinct with higher intensities and without including ventricles.

**Fig. 8.**
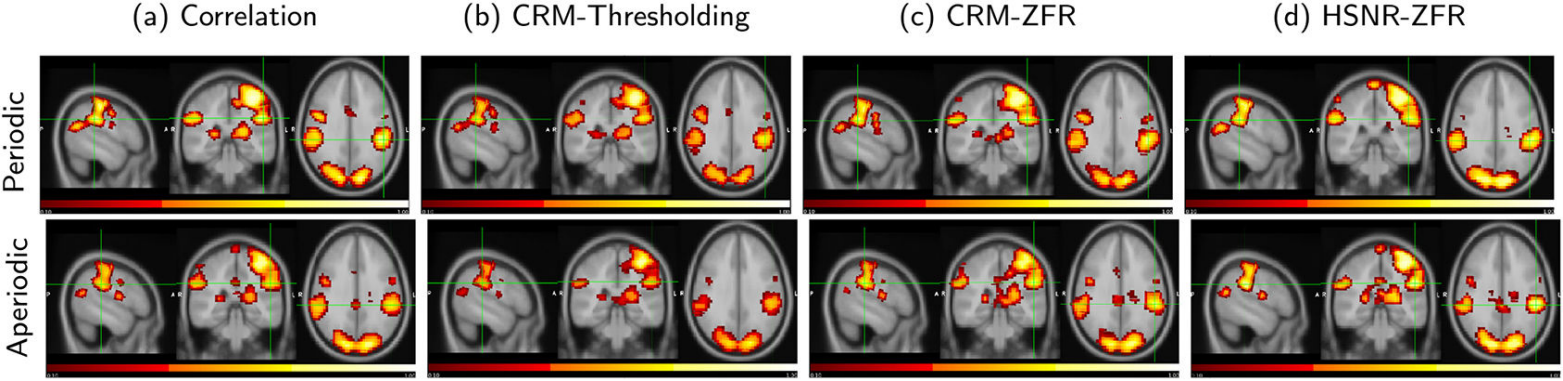
Sagittal, coronal and axial views of the task-related activation map using the stimulus function as the seed for periodic (top row) and random stimuli (bottom row): (a) Correlation map using entire time courses, (b) and (c) CRMs using the estimated onsets by threshold and ZFR respectively and (d) Connectivity map using an average of aggregate correlation in high SNR regions estimated by ZFR. All maps are thresholded (top 20%) and then considered the voxels with clusters consisting of at least 16 neighboring voxels and are finally represented on the same scale (0.1 - 1).

Fig. 8 demonstrates the task-related activation map obtained using the stimulus function as the seed for both the event-based stimuli (periodic and aperiodic) using all four methods namely: correlation, threshold-based CR, ZFR-based CR, and ZFR-based HSNR correlation. The stimulus function was obtained by convolving the actual stimuli with canonical HRF. For both the stimuli, the ZFR-based activation maps (CRM (Fig. 8(c)), HSNR correlation map (Fig. 8(d))) include the motor (supramarginal, precentral, and postcentral gyrus) as well as visual (occipital pole and lateral occipital cortex) area. The Jaccard similarity index between two sessions’ activation maps obtained using four different methods with both the kinds of stimuli presentation has been demonstrated in the Table 2. The similarity is high for periodic stimuli using ZFR-based approaches and presents the robustness of the ZFR-based metrics with less number of time points.

The mean of the score distributions of co-activated and non-co-activated voxels are also illustrated for the two different types of stimuli. Correlation and CR scores (threshold and ZFR) are computed for voxels across the brain using the convolved time course as the seed. The scores for which the target voxels fall inside the active region (Fig. 8) are called co-activation scores and falling outside the region are called non-co-activation scores. A detail of the co-activation and non-co-activation scores for different types of stimuli and for different sessions can be observed in Table 3. The mean (sessions) differences between co-activation and non co-activation scores using the four methods are 0.085, 0.10, 0.155 and 0.175 respectively for the periodic stimuli and are 0.10, 0.13, 0.15 and 0.155 respectively for the aperiodic stimuli. The observed mean difference is more in the case of ZFR-based HSNR correlation for both the kinds of stimuli. It can be seen that the mean co-activation scores are high for the ZFR-based HSNR-driven metric among all cases.

**Fig. 9.**
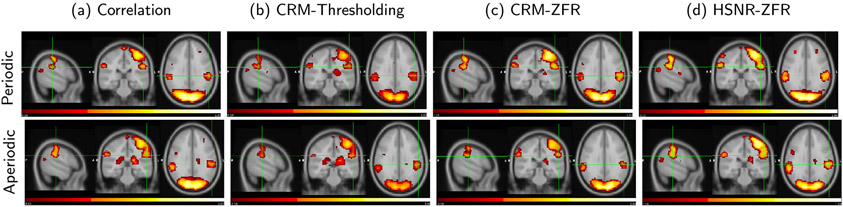
Sagittal, coronal and axial views of static FC using a seed taken from left Primary motor (−53, 0, 22) for periodic (top row) and random stimuli (bottom row): (a) Correlation map using entire time courses, (b) and (c) CRMs using the estimated onsets by threshold and ZFR respectively and (d) Connectivity map using average of aggregate correlation in high SNR regions estimated by ZFR map. All maps are thresholded (top 20%) and then considered the voxels with clusters consisting of at least 16 neighboring voxels and are finally represented on the same scale (0.1 - 1)).

**Fig. 10.**
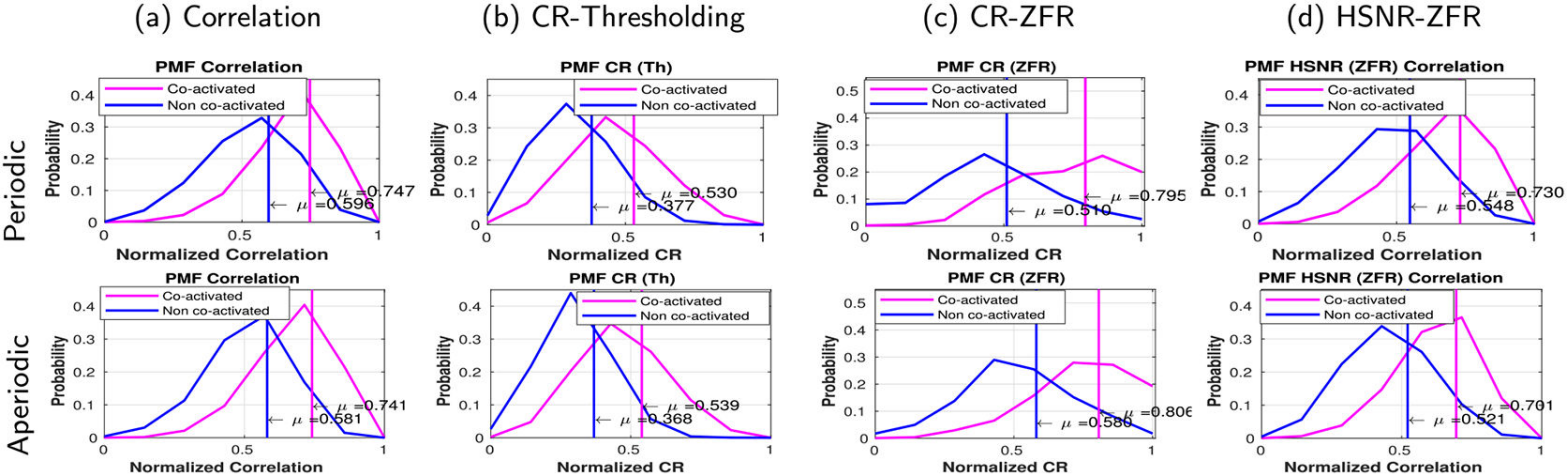
Probability mass function (PMF) of normalized correlation and CR values obtained using the seed (average time course of activated voxels in Fig. 9). The top row shows the co-activated and non co-activated score distributions for periodic stimuli. The bottom row shows the corresponding distributions for the aperiodic stimuli : (a) PMF of correlation for co-activated and non co-activated regions, (b) PMF of CR obtained using thresholding for co-activated and non co-activated regions, (c) PMF of CR obtained using ZFR for co-activated and non co-activated regions and (d) PMF of HSNR (estimated by ZFR) correlation for co-activated and non co-activated regions. Mean differences are (0.15, 0.15, 0.28, 0.18) and (0.16, 0.17, 0.22, 0.18) for periodic and random stimuli respectively.

Corresponding images of Fig. 8 were generated using the actual seed time course from the left sensory-motor cortex (Fig. 9). The spatial maps obtained using the ZFR show the respective task-involved regions as an active areas. The distribution of scores using the four different approaches has been shown in Fig. 10 for periodic and aperiodic stimuli. The average time course of the activated voxels (in Fig. 9) is considered as a seed and connectivity scores are computed using the seed. The scores for which target voxels fall in the active area are called the co-activation scores and falling outside the active region leads to non-co-activation scores. The mean differences between the scores of co-activation and non-co-activation using the four methods are 0.15, 0.15, 0.28 and 0.18 respectively for the periodic stimuli and 0.16, 0.17, 0.22 and 0.18 respectively for the aperiodic stimuli. For both the stimuli, the ZFR-based metrics attain higher differences than the rest of the two and it demonstrates the advantages of ZFR-based metrics for both stimuli.

### 3.3. Efficacy of ZFR on block design working memory data

**Fig. 11.**
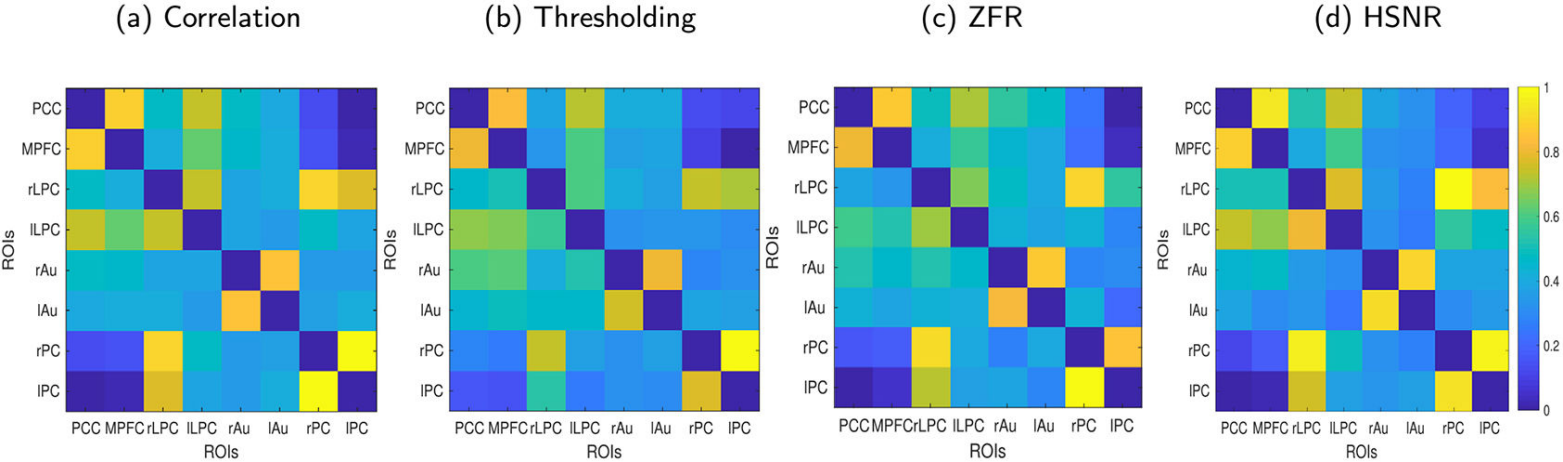
Correlation and conditional rate matrices for eight co-activated and non co-activated regions from three functional networks (DMN, auditory and attention): (a) Correlation matrix considering the entire time courses, (b) and (c) CR matrices considering the estimated onset locations (using threshold and ZFR respectively) and (d) HSNR (six samples around every estimated onset) correlation matrix. Total 8 regions (posterior cingulate cortex (PCC, MNI Co-ordinate: 0, 52, 26), medial pre-frontal cortex (mPFC, MNI co-ordinate: 0, 48, −8), right lateral parietal cortex (rLPC, MNI co-ordinate: 42, −64, 34), left lateral parietal cortex (lLPC, MNI co-ordinate: −46, −64, 34), right auditory cortex (rAu, MNI co-ordinate: 58, −24, 9), left auditory cortex (lAu, MNI co-ordinate: −58, 24, 10), right parietal cortex (rPC, MNI co-ordinate: 38, −48, 34), left parietal cortex (lPC, MNI co-ordinate: −34, −48, 34) are considered.

The efficacy of ZFR based approach is also studied to estimate the temporal location of the BOLD events in complex working memory data. The FCs derived using ZFR-based CR and HSNR correlation have also been discussed. Connectivity matrices among a few selected ROIs using the four different methods are investigated. The working memory task leads to complicated BOLD events in the different brain regions and hence to illustrate the significance of the proposed ZFR-based method, we considered voxels from 8 regions selected manually from three functional networks. Four regions (posterior cingulate cortex (PCC, center MNI coordinate: 0, 52, 26), medial prefrontal cortex (mPFC, center MNI coordinate: 0, 48, −8), right lateral parietal cortex (rLPC, center MNI coordinate: 42, −64, 34), left lateral parietal cortex (lLPC, center MNI coordinate: −46, −64, 34) were selected from default mode network (DMN). Two regions (right auditory cortex (rAu, center MNI coordinate 58, −24, 9), left auditory cortex (lAu, center MNI coordinate: −58, 24, 10)) were taken from auditory (Au) network and two regions (right parietal cortex (rPC, center MNI coordinate: 38, −48, 34), left parietal cortex (lPC, center MNI coordinate: −34, −48, 34)) were taken from attention (Att) network. Average time course of 7 voxels from each of the regions was used for the computation of correlation or CRs between two spatial regions (co-active or non-co-active). Fig. 11 shows the connectivity matrices using four metrics for 8 regions from three functional networks. Pairwise connectivity matrix was first obtained using eight regions with the normalized (min-max) correlation (Fig. 11(a)). Then, a thresholding method using a value of 1.0 (after zero mean unit variance) was used to obtain the corresponding CR matrix shown in Fig. 11(b). The onset estimated using ZFR with a window length of 11 was used to obtain the CR matrix and is shown in Fig. 11(c). Finally, the connectivity matrix obtained using the average of aggregated correlation from the HSNR region was computed (Fig. 11(d)). Following observations can be made:

- The Au and Att networks are significantly connected (*p* < .01) among their integral nodes by all four metrics. However, rLPC has also a strong connection with the Att network (rPC and lPC) by using CR (ZFR) as well as HSNR correlation with a few samples. The connectivity values between rLPC and rPC are 0.90, 0.74, 0.95 and 0.97 using correlation, CR (thresholding), CR (ZFR) and HSNR (ZFR) respectively.
- It should also be noted that the average number of estimated onsets (in 19 subjects) in time courses are 7 and 18 using ZFR and thresholding approaches respectively. So, the CR (ZFR), CR (Threshold), HSNR correlation, and correlation with the entire time course use ~ 7%, ~ 15%, ~ 43% and ~ 100% of the entire BOLD signal to derive the connectivity strength respectively. Although, there was a significant reduction in the number of data points used, overall no significant differences in the connectivity values were observed among the four cases.

**Fig. 12.**
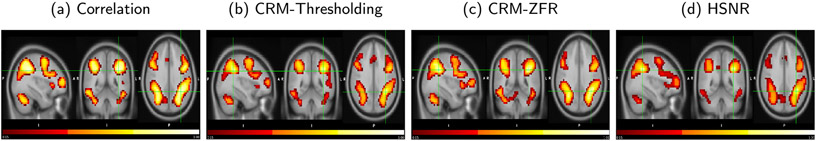
Average of 19 subject’s static FC using a seed (−34, −48, 34): (a) Correlation map using entire time courses, (b) and (c) CRMs using the estimated onsets by threshold and ZFR respectively and (d) Connectivity map using an average of aggregate correlation in high SNR regions estimated by ZFR map. All maps are thresholded (top 20%) and then considered the voxels with clusters consisting of at least 16 neighboring voxels and are finally represented on the same scale (0.15 - 1).

To estimate the FC for working memory data, an active seed was selected from the left parietal lobe (MNI coordinate: −34, −48, 34) and a voxel-wise correlation was performed for the 19 subjects and the average correlation map is shown in Fig. 12(a). Average CRMs of all the subjects using threshold and ZFR are shown in Figs. 12(b) and (c) respectively. The average HSNR correlation map is shown in Fig. 12(d). The ZFR-based connectivity maps show promising spatial extents of activation for working memory task. The numbers of active voxels in the refined maps are 5376, 4785, 4702, and 4635 using correlation, thresholding, ZFR, and HSNR correlation respectively. The ZFR-driven CRM (Fig. 12(c)) can produce a symmetric connectivity map. The aggregated correlation map (in Fig. 12(d)) has less spurious content with preserved spatial symmetricity (e.g. left hemisphere coronal view).

## 4. Discussion

The estimation of the temporal onsets of the BOLD events has always been considered an important problem in the analysis of FC using fMRI. The objective is mostly achieved by choosing the temporal locations, as BOLD events in the time course, which cross a predefined threshold value (Esfahlani et al., 2020; Liu and Duyn, 2013; Tagliazucchi et al., 2012). In this study, we present an approach using ZFR to estimate the temporal onsets of the BOLD events and HSNR regions around the onsets to precisely estimate the FC in task fMRI. Synthetic data and experimental (event-related and block design) data collected from healthy subjects provide promising results in this regard.

It has been demonstrated that a few estimated onset points can be used to reliably generate the connectivity map using ZFR. In previous studies, the hypothesis of using the high values in the BOLD time course is that the triggering of stimuli leads to an increase in blood flow in a specific region. Consequently, the acquired BOLD signal value at the respective temporal location will be relatively high. The estimated FC using the BOLD events suggests that these brief instances can effectively drive functional connectivity. A simple threshold value may not be effective and not always be associated with BOLD events. The ZFR-based approach, on the other hand, exploits the impulse-like characteristics of the NAS and appears to be less prone to the false estimation of BOLD events (Figs. 2, 4 and 5). As was observed in Figs. 3, 8, 9, and 12, the FCs obtained using ZFR were comparable to the maps obtained with the correlation using the entire time courses data set. It should also be noted that estimated BOLD events are only ~ 7% of entire fMRI time points and are able to provide the FC (CRM) akin to the same obtained using the entire time scans. The Jaccard similarity indices between sessions demonstrated the robustness of the ZFR-based metrics like correlation-based metric with entire time points (in Table 2.)

The onsets estimated by the ZFR method are well-matched with the external stimuli with ~ 2 sec delay (Figs. 4 and 5) for both periodic and aperiodic event-related design. The obtained sensitivity and specificity scores (Table 1, Fig. 7) show that the ZFR-based method outperforms the thresholding method and excludes the spatial spurious content (ventricles) in its maps. The estimated FC maps using all the methods show relatively less noisy spatial components in the case of periodic stimuli than the random stimuli. The ZFR-based two features estimate the task-related activation maps extending to fewer ventricles in the brain for both types of stimuli (Fig. 8). The mean of the distributions for co-activation and non-co-activation scores using the stimulus function as the seed are well separated for the ZFR-based approaches (see Table 3) indicating two useful metrics. Fig. 10 shows that the difference in mean of the distributions attains the highest for ZFR-based CR and HSNR correlation using the average time course of active voxels as the seed. The observed difference is highest for the CR-ZFR among all for periodic stimuli with fewer estimated onset points. In simulated data, the noise level is high (can be seen in Fig. 2) in comparison to the experimental data. As a result, ZFR derives a greater number of estimated onsets in the time course than the estimated onsets in the experimental event-related data. But, the overall true positives (TP) and true negatives (TN) are more in the case of ZFR than the thresholding.

Using the proposed method, we could reliably estimate the temporal onsets of the BOLD events without a priori information regarding the stimulus timing. The estimated BOLD events matched with the timing of stimuli presented with a delay. This would provide important information in the studies where the exact onset time is not known. In several clinical applications like stroke or traumatic injury, hallucination, and even in healthy subjects having habituation, a method like this would be useful in the accurate identification of the task-induced signal changes.

The region around the high valued time points in the BOLD signal has HSNR and helps in estimating reliable FC (Buckner and Randy, 1998; Li et al., 2014). The transient noise may also induce a substantial effect on FCs (Hutchison et al., 2013; Liu et al., 2018). The ZFR can extract the temporal location of the BOLD events but around the onsets has relatively HSNR in the BOLD response and use of the same helps in the reliable estimate of FC by reducing the transient noise. The ZFR can also capture the BOLD events in the time course for block design fMRI. To obtain a precise estimate of FC, in the case of block design, the HSNR regions are used and the obtained FC shows good spatial extent with less spurious contents (Fig. 12(d)). Here, the ZFR-driven HSNR segment in the BOLD response considers a small number of time points to estimate the FC by mitigating the random effect of noise events.

The conditional rate matrix (Fig. 11) illustrates that ZFR-driven CR establishes co-activation of rLPC (an integral part of DMN) with Att network (rPC and lPC) whereas, for the CR (threshold), it is not so vivid. Previous studies have reported significant correlations between these regions in different phases of working memory task (Piccoli et al., 2015).

For the computation of CR, we have used a delay of at most two-time points for matching the estimated onsets in target time course with the seed onsets based on prior work (Tagliazucchi et al., 2011). It should be noted that for this study, where a TR of 2sec was used, 2 data points may be adequate for this approach. However, for shorter TR, perhaps more data points may be needed. ZFR uses an optimal window in its local mean subtraction step. Choosing a suitable window is crucial and hence a shortcoming to estimate the optimal number of onsets as it depends on the sampling rate (TR) as well as the experimental paradigm. If we choose a small window for ZFR it may result in spurious onsets (False positives) on the other hand if we take a long window it may miss the actual onsets (False negative). We have empirically varied it around length which we can get from the idea of average samples in the BOLD time course for an event to occur (due to stimulus) and chose the optimal size of the window. We have set the window size 15 and 11 for the event-related design and the block design task respectively. The second parameter used in determining the HSNR feature in this study was set at 6 data points around every estimated onset. This parameter can also be determined by considering a few samples starting from 3 or from knowledge of the typical ON time of the stimulus in event design or from the block experiment where the onset time is at least 8 seconds and the off period is at least 10 seconds. A formal analysis and a reliability test have been demonstrated in the supplementary section for the optimal HSNR window size.

## 5. Conclusions and future directions

The current manuscript presents a novel way to extract the temporal onsets of the BOLD events in fMRI time courses using the ZFR followed by extraction of HSNR regions. The CRM, estimated by the ZFR-driven BOLD events, with respect to a particular seed voxel underpins the corresponding functional organization of finger tapping and working memory task data. The neuronal activity inducing information in the HSNR regions selected by the ZFR approach can also drive well spatially located FCs with less spurious content for both tasks. Results from this study demonstrated that a few numbers of inflection points (less than 7%) denoted as the BOLD events and important segments in the BOLD time course denoted as the HSNR regions (less than 43%) have the ability to produce respective FCs in task fMRI without any prior knowledge of EP. The ratio of mean percentage signal change within HSNR regions to the mean percentage signal change in non-HSNR regions could be a potential measure of data quality especially when noise (e.g. thermal noise) is higher. The work will be extended in the future in estimating neuronal activity signal and FC for resting-state fMRI by extracting the information of spontaneous BOLD events and also in different pathological conditions.

## Supplementary Material

1

## Figures and Tables

**Table 1:** Average sensitivity and specificity of the estimated onsets of neuronal events in the BOLD time courses from 5 subjects for finger tapping task.

Method	Sensitivity	Specificity
Thresholding (Periodic)	0.29	0.88
ZFR (Periodic)	0.52	0.95
Thresholding (Aperiodic)	0.26	0.87
ZFR (Aperi)	0.49	0.95

**Table 2:** Jaccard similarity distances between the connectivity maps obtained in two sessions. Here S, Per and Aper stand for session, periodic and aperiodic respectively.

Metric	Per S1 - Per PS2	Aper S1 - Aper S2
Correlation	0.45	0.74
Thresholding	0.45	0.73
ZFR	0.46	0.65
ZFR-High SNR	0.50	0.74

**Table 3:** Mean co-activation and non-co-activation scores using four different metrics.

Session (Stimuli)	Metric	Co-activation score	Non-co-activation score	Difference
Session 1 (Periodic)	Correlation	0.54 ± 0.17	0.44 ± 0.15	0.10
	CR (Threshold)	0.42 ± 0.16	0.35 ± 0.12	0.07
	CR (ZFR)	0.50 ± 0.20	0.33 ± 0.18	0.17
	Correlation (High SNR-ZFR)	0.70 ± 0.16	0.51 ± 0.15	0.19
Session 2 (Periodic)	Correlation	0.51 ± 0.18	0.45 ± 0.13	0.07
	CR (Threshold)	0.44 ± 0.13	0.31 ± 0.13	0.13
	CR (ZFR)	0.48 ± 0.18	0.34 ± 0.17	0.14
	Correlation (High SNR-ZFR)	0.62 ± 0.18	0.46 ± 0.15	0.16
Session 1 (Aperiodic)	Correlation	0.55 ± 0.19	0.48 ± 0.15	0.07
	CR (Threshold)	0.40 ± 0.17	0.33 ± 0.14	0.07
	CR (ZFR)	0.40 ± 0.27	0.28 ± 0.22	0.11
	Correlation (High SNR-ZFR)	0.61 ± 0.18	0.50 ± 0.15	0.11
Session 2 (Aperiodic)	Correlation	0.57 ± 0.17	0.44 ± 0.17	0.13
	CR (Threshold)	0.45 ± 0.22	0.26 ± 0.13	0.19
	CR (ZFR)	0.46 ± 0.25	0.27 ± 0.19	0.19
	Correlation (High SNR-ZFR)	0.64 ± 0.17	0.44 ± 0.13	0.20

**Fig. A1.**
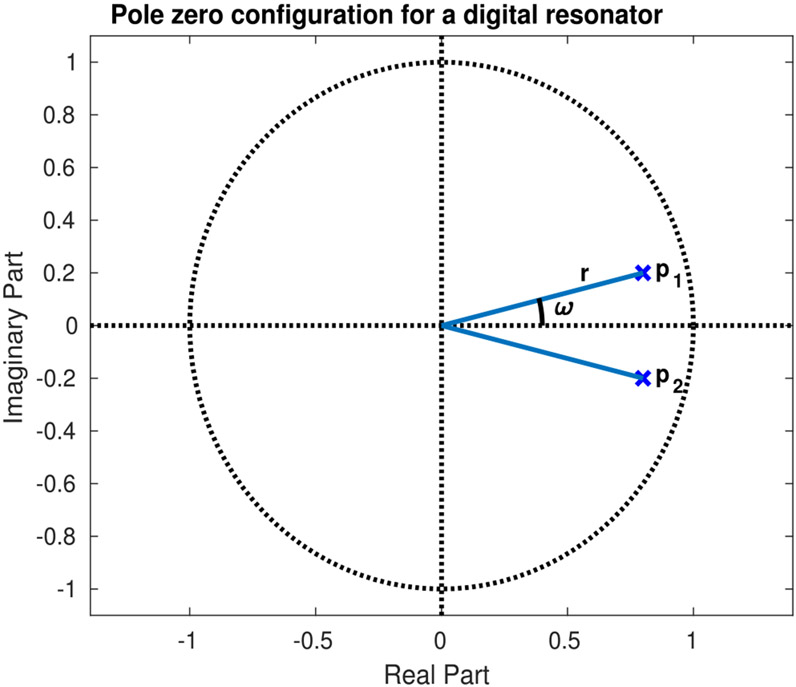
Pole-zero configuration for a digital resonator whose resonant frequency is *ω*.

**Fig. A2.**
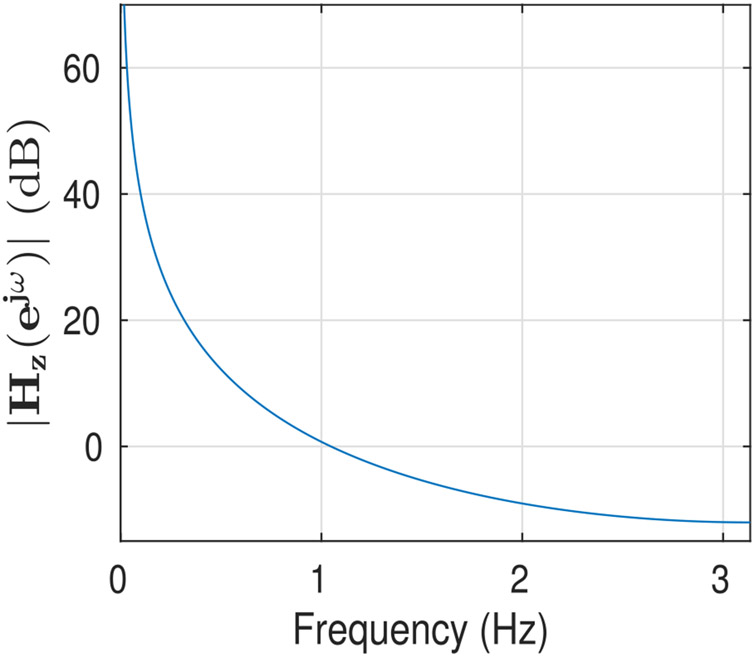
Frequency response (∣*H_z_*(*e*^*jω*^)∣) of the ZFR.

## Data Availability

Data will be made available on request.

## Footnotes

1
http://www.fil.ion.ucl.ac.uk/spm/

## Appendix A. Zero frequency resonator

A digital resonator is an infinite impulse response (IIR) system (linear) having a complex conjugate pair of poles located inside the unit circle of the z-plane. The angle (*ω*) of the poles with abscissa (*R*_*e*_(*z*)) of the z-plane decides the resonant frequency of the resonator, while the distance of the poles in the unit circle sets the bandwidth. The closer they are to the unit circle, the smaller the bandwidth. The transfer function (*H*_*z*_(*z*)) of a linear system having a pair of complex conjugate poles at *r* cos(*ω*) ± *jr* sin *ω* (see Fig. A.13) (Oppenheim, 1999),

(A.1)
$$H_{z}(z)=\frac{1}{\left(1-(r\;\cos\;\omega +jr\;\sin\;\omega )z^{-1})(1-(r\;\cos\;\omega -jr\;\sin\;\omega )z^{-1}\right)}$$

(A.2)
$$=\frac{1}{1-2r\;\cos\;\omega z^{-1}+r^{2}z^{-2}}$$

For an ideal resonator (minimum width) with zero resonant frequency, the value of *r* and *ω* would be one and zero respectively. Thus transfer function of the resonator is

(A.3)
$$H_{z}(z)=\frac{1}{1-2z^{-1}+z^{-2}}.$$

Fig. A.14 demonstrates the frequency response (∣*H_z_*(*e*^*jω*^)∣) of the ZFR. It can be observed that it is a low pass filter.
