# Supplementary material for: Estimation of neuronal task information in fMRI using zero frequency resonator
Source: Neuroimage. Author manuscript; Available in PMC 2023 Nov 9. (PMC10635735; doi:10.1016/j.neuroimage.2023.119865)
Supplement: 1 [file NIHMS1867085-supplement-1.docx]

**Supplemental Material**

**Estimation of Neuronal Task Information in fMRI using Zero Frequency Resonator**

Sukesh Kumar Das, Anil K. Sao, Bharat Biswal

1. **Determination of the length of the HSNR segment:**

A reliability test was performed to find an optimal sample size for the HSNR segment. We considered 3 out of 5 subjects and got 10 combinations in 10 iterations for each of which we estimated the average HSNR correlation map. These 10 connectivity maps yield 45 (=10*(10-1)/2) Jaccard similarity distances (JSD). For different lengths of high SNR samples, we have computed the distances (between maps) as shown in Supplementary Figure 1. We have also shown the variances using the different lengths of the HSNR segment in Supplementary Table 1. It shows that length 6 (-1 0 1 2 3 4) shows comparatively less variance in JSD along the different combinations of iterations.


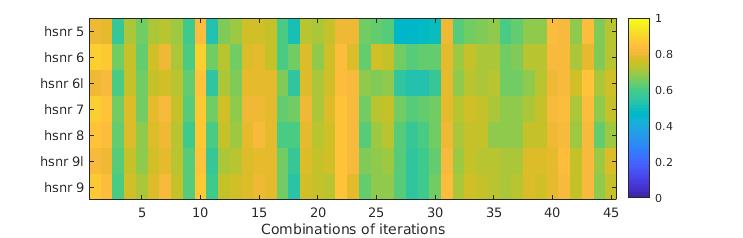


**Supplementary Figure 1**. JSD between the connectivity maps. 10 iterations give 45 distances for each HSNR length.

**Supplementary Table 1. Variation of JSDs**

| **Length (samples location around onsets, estimated onset location is on 0)** | **Variance** |
| --- | --- |
| hsnr 5 (-1 0 1 2 3) | 0.102 |
| hsnr 6 (-1 0 1 2 3 4) | 0.075 |
| hsnr 6l (-2 -1 0 1 2 3) | 0.088 |
| hsnr 7 (-2 -1 0 1 2 3 4) | 0.076 |
| hsnr 8 (-2 -1 0 1 2 3 4 5) | 0.083 |
| hsnr 9l (-3 -2 -1 0 1 2 3 4 5) | 0.078 |
| hsnr 9 (-2 -1 0 1 2 3 4 5 6) | 0.083 |
